# Self-Reported Diabetes in Older Adults: A Comparison of Prevalence and Related Factors in the Mexican Health and Aging Study (2015, 2018, and 2021)

**DOI:** 10.1155/2024/2527791

**Published:** 2024-08-12

**Authors:** Alvaro García Pérez, Teresa Villanueva Gutiérrez, Laura Bárbara Velázquez-Olmedo

**Affiliations:** ^1^ Faculty of Higher Studies (FES) Iztacala National Autonomous University of Mexico (UNAM), Mexico City, Mexico; ^2^ Health Care Department Metropolitan Autonomous University-Xochimilco, Mexico City, Mexico; ^3^ Faculty of Dentistry National Autonomous University of Mexico (UNAM), Mexico City, Mexico

**Keywords:** health insurance coverage, multimorbidity, older adults, rural, self-reported diabetes

## Abstract

**Aim:** To estimate the prevalence and factors associated with diabetes among older adults and compare the prevalence rate of a three-round national survey of the Mexican Health and Aging Study (MHAS).

**Methods:** A cross-sectional study was conducted with data obtained from MHAS 2015 (*n* = 8167), 2018 (*n* = 7854), and 2021 (*n* = 8060), which comprised a nationally representative sample of older adults in Mexico. The measures included sociodemographic characteristics and health. A binary logistic regression model was used to identify the association between independent variables and self-reported diabetes.

**Results:** The prevalence of diabetes was 26.3%, 27.7%, and 28.1% in 2015, 2018, and 2021, respectively. This prevalence decreased with age and was higher for female, urban older adults, those with multimorbidity, a lower level of education, and without social security coverage for the three years. Age was associated with a lower possibility of presenting diabetes ([OR = 0.79[0.71–0.89]] and [OR = 0.41[0.33–0.52]] in groups aged 75–84 years and ≥85 years, respectively). Females continue to be more likely to present diabetes than males (OR = 1.39 [95% CI 1.25–1.55]). Older adults living in rural areas are 20% less likely to present diabetes than those living in urban areas (OR = 0.80 [95% CI 0.69–0.93]). Uninsured older adults (OR = 1.35 [95% CI 1.20–1.53]), those who wear glasses (OR = 1.23 [95% CI 1.16–1.30]), those with multimorbidity (OR = 1.13 [95% CI 1.01–1.27]), and those who currently drink alcohol (OR = 1.12 [95% CI 1.00–1.25]) were significantly more likely to have diabetes.

**Conclusion:** An elevated prevalence of diabetes was found in older adults in Mexico, while not having access to social security was associated with a higher possibility of presenting diabetes and living in a rural area was associated with a lower possibility of presenting diabetes. Detection, prevention, and control programs should be implemented to reduce the incidence and severity of the disease in older adults and, thus, prevent its associated complications.

## 1. Introduction

The age of the global population is continuing to rise due to increased life expectancy and the evolution of demographic factors such as fertility and mortality. The aging of the population is one of the major challenges to public health, given that people are living longer, while both the proportion and number of older people in the total population are rapidly increasing [1]. The World Health Organization has predicted that the number of people aged 60 years and over will increase from over 1000 million in 2020 to 1400 million in 2030 and 2100 million by 2050 [2]. Similarly, life expectancy has increased, going from 66.8 years in 2000 to 73.4 years in 2019 [3].

Population aging is one of the main demographic phenomena presenting in Latin America and the Caribbean, with the proportion of adults aged 60 years and over increasing to 25%–30% by 2050 [4]. In Mexico, the number of adults aged 60 years and over will nearly triple from 6.3% in 2010 to nearly 23% by 2050 [5]. Because various health problems both become more common and worsen with age, there is evidence that older adults may experience various diseases at the same time. Similarly, it has also been observed that most chronic conditions are associated with aging [6].

Diabetes mellitus is a chronic metabolic condition characterized by hyperglycemia and high levels of glycosylated hemoglobin [7]. According to the International Diabetes Federation (IDF), approximately 537 million people aged 20–79 years old have diabetes at a global level, and, according to projections for the same age range, a respective 643 and 783 million adults will be living with diabetes around the world by 2030 and 2045 [8]. In Mexico, results obtained from the 2018 and 2020 *Encuesta Nacional de Salud y Nutrición* (ENSANUT, or the National Health and Nutrition Examination Survey) show prevalences of diabetes of 34.0% and 28.1%, respectively, in adults aged 60–69 years [9].

Previous research has estimated that the prevalence of diabetes in Mexico will continue to increase [10, 11], because one in two children will develop diabetes in the future due to weight, lack of physical activity, and other factors related to public health change [12].

While aging may be the main cause of the increased prevalence of diabetes [13], this is also due to the prevalence of obesity associated with unhealthy lifestyle changes and industrialized diets [14]. Moreover, the presence of comorbidities, a higher predisposition to hypoglycemic events, smoking, and social inequality [15] all contribute to the increased prevalence of the disease in older adults.

Type 2 diabetes is one of the main public health problems in Mexico and is one of the main causes of premature disability, blindness, end-stage renal disease, and nontraumatic amputations. It is also one of the 10 most common causes for the hospitalization of Mexican adults and generates a high level of direct and indirect costs for treating the complications of the disease, such as medical appointments, diagnoses, and hospitalizations [16]. Therefore, estimating the prevalence of diabetes and ascertaining the risk factors for the condition will assist in the identification of populations at a greater risk of presenting microvascular and macrovascular complications, a worse quality of life, and premature mortality. This will also enable updated estimates for the epidemiology of diabetes in older Mexican adults. Finally, it would aid in the development of policies for the prevention, management, and treatment of the disease. Therefore, the present study is aimed at estimating both the prevalence of diabetes in 2021 and the factors associated with the condition, in order to then compare this prevalence with that found in representative surveys conducted at a national level for older adults by the Mexican Health and Aging Study (MHAS) in 2015 and 2018.

## 2. Material and Methods

The MHAS is a national longitudinal study of adults aged 50 years and older. The baseline evaluation was carried out in 2001 and comprises five rounds (2003, 2012, 2015, 2018, and 2021) of a nationally representative prospective study of adults born before 1951 and is representative of both urban and rural environments. The surveys are conducted under the supervision of coordinators from both the United States and Mexico, while MHAS is partially funded by the National Institutes of Health/National Institute of Aging and the *Instituto Nacional de Estadística y Geografía* (INEGI or National Institute of Statistics and Geography). The data files and documentation are available for public use at https://enasem.org/Home/index_esp.aspx. The thematic content of the MHAS survey included the following: demographic data, the number of residents of a household and children's rosters, self-reported health in various dimensions (chronic diseases, physical function, perceived global health, depression, and cognition), institutional support, life satisfaction, use of time, social support and social engagement, housing conditions, and economic aspects, such as health expenditure, health insurance coverage, pensions received or expected, income by sources, and the value of accumulated assets.

### 2.1. Ethics Approval and Consent to Participate

The research carried out by the present study on the MHAS databases adhered to the relevant ethical criteria for research on human subjects and was approved by the corresponding ethics committee (University of Texas Medical Branch and, in Mexico, INEGI and the National Institute of Public Health) (NIH R01AG018016). Informed consent was obtained in writing from all participants.

### 2.2. Study Design

This cross-sectional study used data from MHAS 2015, 2018, and 2021.

### 2.3. Study Population

The study was conducted on a subsample of 24,081 adults aged 65 years and over, which, by year, corresponded to 8167 older adults in 2015, 7854 in 2018, and 8060 in 2021. The inclusion criteria applied by the present study for selecting participants were being an adult over the age of 65 years, of either gender, and who did not present missing data in the database. The exclusion criteria were being an older adult who did not provide signed informed consent or who refused to participate in the study.

### 2.4. Independent Variables

The following variables were divided into the following categories: age (65–74 years, 75–84 years, and ≥85 years); gender (male/female); marital status (without spouse/partner and with spouse/partner); years of education, which was used to compare those adults who had completed 9 years of formal education or more with those who had completed less than 9 years (corresponding, in Mexico, to primary and secondary school combined) (no education, 1–9 years, and ≥10 years); currently a smoker (yes/no); currently drinks alcohol (yes/no/has never drink alcohol); wears glasses (yes/no); uses an auditory device (yes/no); number of dental visits in the last year (no/≥1 visit); has he/she seen a doctor or medical personnel in the last 2 years (no/≥1 visit); and place of residence (urban/rural). In Mexico, a rural locality is classified as having a population of less than 2500 inhabitants.

Self-reported health was evaluated by means of a standardized question asking, “Would you say your health is...?” with the responses ranked on a Likert scale and categorized into *excellent/very good*, *good*, *fair*, and *poor*. The evaluation of self-reported multimorbidity corresponds to a number of chronic diseases, such as hypertension, heart attack, asthma, arthritis, and cancer, and is considered a disease or condition as present if the participant answered “yes” to the question “Has a doctor or medical personnel ever diagnosed you with...?” The answers were then grouped into nonmultimorbidity and multimorbidity ≥2 chronic conditions.

The present study defined health insurance coverage as being in receipt of insurance coverage under the following programs: *Instituto Mexicano del Seguro Social* (IMSS or Mexican Institute of Social Security); *Instituto de Seguridad y Servicios Sociales de los Trabajadores del Estado* (ISSSTE or Institute of Security and Social Services for State Workers); *Petroleos Mexicanos* (PEMEX or Mexican Petroleum); *Secretaria de Defensa Nacional* (SEDENA or Ministry of Defense); and the *Secretaria de Marina* (SEMAR or Ministry of the Navy) programs. Being uninsured was defined by the receipt of healthcare via the *Instituto Nacional de Salud para el Bienestar* (INSABI or National Health Institute for Welfare) or either having or not having private insurance (insured/uninsured).

### 2.5. Dependent Variable: Self-Reported Diabetes

Older adults were classified as having self-reportedly diagnosed diabetes if they answered “yes” to the question, “Has a doctor or another medical practitioner ever diagnosed you with diabetes?”

### 2.6. Statistical Analysis

All analyses were conducted using the Stata 15 software (Stata Corp., College Station, TX, United States).

Descriptive statistics (frequencies and percentages) were used to summarize the characteristics of the study population, while the study also used databases corresponding to the older adult population identified by MHAS 2015 (*n* = 8167), MHAS 2018 (*n* = 7854), and MHAS 2021 (*n* = 8060). The prevalences were obtained along with their respective 95% confidence intervals (95% CI), while a trend test was conducted to estimate whether the increased prevalence of diabetes was statistically significant over the course of the three national surveys (2015, 2018, and 2021). The trend test used was a Poisson regression, with the dependent variable being self-reported diabetes and the independent variable being the year of the survey, adjusted for age and gender. The association between the dependent variable self-reported diabetes (yes/no) and the independent variable was tested via a binary logistic regression model adjusted for confounders [Li = *B*_0_ + *B*_1_*X*_1_ + *B*_2_*X*_2_ + *B*_3_*X*_3_+⋯+*B*_*k*_ *X*_*ki*_], with the odds ratio calculated to a 95% CI, with values of *p* ≤ 0.05 considered statistically significant. Model diagnostic tests were conducted using the Hosmer–Lemeshow goodness-of-fit test. The analysis used complex sample analysis procedures via the SVY commands in Stata 15. A two-way interaction analysis between all the independent variables in the model was carried out, with no significant interaction found (*p* > 0.05).

## 3. Results

Table 1 presents the demographic and health characteristics of the older adults considered in the present study. In total, the sample of 24,081 older adults had an average age of 74.4 (±7.13) years and comprised similar percentages of men and women and a higher proportion of older urban than older rural adults. More than half of those surveyed were married or had a partner and used glasses.

On the other hand, an increase in both the number of doctor's visits and social security coverage was observed. Finally, a third of the older adults presented comorbidities in all three periods.

**Figure 1:**
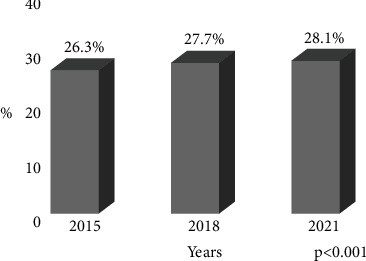
Prevalence of self-reported diabetes among older adults of Mexican Health and Aging Study (MHAS 2015, 2018, and 2021) (*n* = 24,081). Trend test for the prevalence of self-reported diabetes, adjusted for age and sex PR = 1.013, *p* = 0.006.

The prevalence of self-reported diabetes was 26.3% (95% CI 25.3–27.2), 27.7% (95% CI 26.7–8.7), and 28.1% (95% CI 27.0–29.0) in the years 2015, 2018, and 2021, respectively (Figure 1). The prevalence of self-reported diabetes was found to have increased with a positive trend (*p* = 0.006). In 2021, the prevalence across the three age groups was higher than that observed in 2015. Comparing the results obtained for 2021 and 2015, the prevalence of diabetes was higher among the women than among the men sampled. The prevalence of self-reported diabetes in urban locations was higher than that found for rural locations: 27.5% versus 21% in 2015, 29.1% versus 22.1% in 2018, and 29.4% versus 22.7% in 2021. A third of the older adults with diabetes also presented another comorbidity in 2015, 2018, and 2021. Finally, the prevalence of diabetes in uninsured subjects who use glasses and consume alcohol increased in all three study periods (Table 2).

### 3.1. Multivariable Analysis

A binary logistic regression model was generated to identify the conditions associated with self-reported diabetes in older Mexican adults (Table 3). Eight variables were considered in the binary logistic regression analysis. Subjects aged 75–84 years (OR = 0.79 [95% CI 0.71–0.89]) and ≥85 years (OR = 0.41 [95% CI 0.33–0.52]) were less likely to present diabetes than those aged 65–74 years, while females continued to be more likely to present diabetes than males (OR = 1.39 (95% CI 1.25–1.55]). Older adults living in rural areas are 20% less likely to have diabetes than older adults living in urban areas (OR = 0.80 [95% CI 0.69–0.93]). Uninsured older adults are 35% more likely to present diabetes (OR = 1.35 [95% CI 1.20–1.53]) than those older adults with social security coverage. Other variables, such as using glasses (OR = 1.23 [95% CI 1.16–1.30]), multimorbidity (OR = 1.13 [95% CI 1.01–1.27]), and currently drinks alcohol (OR = 1.12 [95% CI 1.00–1.25]), were associated with the presence of diabetes in older adults.

## 4. Discussion

The present study found that the prevalence of diabetes, in accordance with MHAS 2015, 2018, and 2021, was 26.3%, 27.7%, and 28.1%, respectively. This finding corresponds to an increase of 1.8% from 2015 to 2021. Generally, the prevalence of diabetes was similar to that reported by Basto-Abreu et al. and Campos-Nonato et al., who, using data obtained from ENSANUT 2016, 2018, and 2020 for adults aged 60 years or more, found a prevalence of 27.7%, 34.0%, and 28.1%, respectively [9, 17]. A 17.6% and 20.1% prevalence of diabetes was found for older Brazilian adults in 2003 and 2008, respectively [18]. The increased prevalence of diabetes in older Mexican adults may be due to the increased number of diagnosed cases of diabetes, and previous cross-sectional studies using Mexican national surveys indicate that the prevalence of diabetes mellitus is increasing: 7.5% in 2000, 9.2% in 2012, 13.7% in 2016, 14.7% in 2018, and 16.9% in 2021 [19]. Similarly, other factors related to the increase in the prevalence of diabetes could be population growth, aging, urbanization, and unhealthy lifestyle changes that cause an increase in the number of people with diabetes.

It was also observed that, from 2015 to 2021, the largest increase in the prevalence of diabetes was observed for the 65–74 year group, with the prevalence of diabetes decreasing as age increased over that 6-year period. Similar results were found in the *Encuesta sobre Salud, Bienestar y Envejecimiento* (SABE or National Survey of Health, Wellbeing, and Aging) for adults over the age of 60 years in Ecuador [20]. Of the age groups considered, the present study found the highest prevalence of diabetes in the 60 years and over group and the lowest in those aged 85 years and over. It is likely that this difference will continue to be present in 2030, although less marked, given that the average age of the Mexican population is 75.4 years [21]. Furthermore, it is possible that the increased number of people with diabetes or long-term diabetes alters the profile of the disease in different populations at a global level, particularly due to a higher incidence of complications specific to diabetes [22].

According to MHAS 2021, the risk factors associated with diabetes were age, gender, place of residence, use of glasses, social security coverage, multimorbidity, and alcohol consumption. Women continue to present a higher possibility of presenting diabetes than men. Moreover, the results of the SABE survey show a higher prevalence of diabetes in women than in men (19.7% vs. 12.9%, respectively) [20]. On the other hand, Nordström et al. found, in adults over the age of 70 years, that the prevalence of diabetes was higher in men than in women (14.6% vs. 9.1%, respectively) [23]. The difference found between gender and the prevalence of diabetes could be explained why in middle-aged populations, men show a higher prevalence of diabetes than women. However, postprandial hyperglycemia increases substantially in women as they age, contributing to an increase in the prevalence of undiagnosed diabetes in women after the age of 60 [24]. At the same time, the increasing prevalence of diabetes with age combines with the higher number of older women than men in the majority of populations to, thus, affect these differences as well [24]. Studies have described how impaired fasting glucose and impaired glucose tolerance present differences in terms of gender in all the populations studied [25]. The present study found that older rural adults are less likely to present with diabetes than older urban adults. In fact, the increase in the prevalence of diabetes has been documented at higher levels in urban areas [26]. Although urbanization is a significant phenomenon closely linked to modernization, socioeconomic status, and lifestyle, it is also associated with an increased incidence of diabetes. According to the IDF 2021, the prevalence of diabetes was higher in urban than rural areas (12.1% vs. 8.3%, respectively) [27]. A meta-analysis conducted for the years 1990–2012 found that, while the prevalence of diabetes has increased in all the rural areas of the world, this increase occurred more quickly in middle- to low-income rural areas than in high-income areas [28].

It is estimated that, in Mexico, 21% of the population live in rural areas [29], with older rural adults tending to be both socially and geographically isolated, to live on limited resources, and to experience difficulties in accessing and paying for healthcare services, which can lead to health problems going untreated [30]. It is known that, when the condition begins to manifest, around 30% of people living with diabetes have little knowledge of their condition/are not aware of their condition, a percentage that increases in rural areas, generating health complications if it is not detected and treated in time [31]. In light of this scenario regarding diabetes, the early detection of risk factors would enable the implementation of preventive strategies that aim to reduce the prevalence of the disease. Furthermore, knowledge about diabetes may play an important role in motivating both urban and rural populations to apply measures to both prevent the disease and reduce its complications.

The present study found that older adults without social security coverage are more likely to present with diabetes than those with social security or health insurance coverage. It also found that social security coverage increased from 57.6% to 64.3% over the period sampled. For some time, the Mexican healthcare system has made efforts to increase access to healthcare services for both those affiliated with the social security program and those who are not, in order to widen coverage and ensure equitable access to healthcare services, thus reducing social inequality and extreme poverty. Healthcare coverage is important not only for helping adults diagnosed with diabetes manage their condition but also for those with undiagnosed diabetes, an important consideration given that, according to ENSANUT 2022, approximately 6.7% of adults aged 60 years and over have undiagnosed diabetes [32]. The older adults considered by the present study not to have social security coverage are more likely to present serious complications and high costs for medical treatment, given that the diagnosis of their condition is likely to be delayed due to infrequent medical appointments.

Multimorbidity and alcohol consumption are associated with the presence of diabetes. For example, Quiñones, Markwardt, and Botoseneanu reported that people with multimorbidity and diabetes are more likely to develop physical and functional limitations and a greater risk of developing mental health problems that could interfere with their lifestyle and adherence to a medication regime [33]. Alcohol consumption is a risk factor for diabetes. According to the literature, the relationship between alcohol consumption and diabetes is inconsistent. Several studies have reported that moderate alcohol consumption is associated with a decrease in diabetes, while high alcohol consumption is associated with an increased risk [34]. Moderate alcohol consumption has a protective effect against the development of diabetes, given that it increases insulin sensitivity [35]; however, Engler, Ramsey, and Smith found that excessive alcohol consumption is a barrier to diabetes self-care and can also affect the clinical course of the condition [36].

The government of Mexico has designed national initiatives to address the growing burden posed by diabetes. However, our research suggests that the prevalence of diabetes will continue to rise in the coming years among older adults. The government of Mexico, together with the National Institute of Public Health, has developed national policies to prevent obesity and diabetes and promote healthy nutrition in the population. In addition to these federal efforts, there are several state programs to prevent diabetes. IMSS, ISSSTE, and other public health services such as INSABI have developed their own national programs to prevent and control diabetes, both for the insured and uninsured population [37, 38].

### 4.1. Limitations

One of the limitations of the present study is that it used cross-sectional surveys (MHAS 2015, 2018, and 2021), which made it impossible to make causal inferences. Another limitation is that the present study does not identify the different types of diabetes in the population; therefore, it could affect the results obtained. On the one hand, no differentiation was made between heavy and light drinkers and smokers. On the other hand, one of the advantages of the study is that it used a representative sample of older adults in Mexico, with both urban and rural representation, which enabled the prevalence of diabetes to be calculated. The prevalence of diabetes was determined via self-reporting in the present study. It has been observed that self-reporting is a good indicator of a diagnosis significantly associated with mortality and the health of the population in general [39]. But generally, self-reported information has a recall bias.

## 5. Conclusions

An elevated prevalence of diabetes was found in older adults in Mexico, while not having access to social security was associated with a higher probability of presenting diabetes and living in a rural area was associated with a lower probability of presenting diabetes. Diabetes is a chronic metabolic disease that has an economic impact on both patients and healthcare services. Detection, prevention, and control programs should be implemented to both reduce the incidence and severity of the disease in older adults and prevent complications, in order to exert a positive effect on quality of life.

## Figures and Tables

**Table 1:** Sociodemographic and health characteristics of older adults of Mexican Health and Aging Study (MHAS 2015, 2018, and 2021).

**Variable**	**2015 (** **n** = 8167**)**		**2018 (** **n** = 7854**)**		**2021 (** **n** = 8060**)**	
	**n**	**%**	**n**	**%**	**n**	**%**
Age						
65–74 years	4980	61.0	4466	56.9	4326	53.7
75–84 years	2457	30.1	2577	32.8	2825	35.0
≥85 years	730	8.9	811	10.3	909	11.3
Gender						
Male	3794	46.5	3514	44.7	3481	43.2
Female	4373	53.5	4340	55.3	4579	56.8
Marital status						
Without spouse/partner	3461	42.4	3294	41.9	3623	44.9
With spouse/partner	4706	57.6	4560	58.1	4437	55.1
Place of residence						
Urban	6627	81.1	6303	80.3	6440	79.9
Rural	1540	18.9	1551	19.7	1620	20.1
Years of education						
No education	1886	23.1	1676	21.3	1488	18.5
1–9 years	5460	66.8	5377	68.5	5601	69.5
≥10 years	821	10.1	801	10.2	971	12.0
Currently smoker						
No	7434	91.0	7212	91.8	7398	91.8
Yes	733	9.0	642	8.2	662	8.2
Currently drinks alcohol						
No	5725	70.1	1643	20.9	1746	21.7
Yes	1570	19.2	5174	65.9	4899	60.8
Never drank alcohol	870	10.7	1037	13.2	1415	17.5
Wear glasses						
No	3983	48.8	3397	43.2	3420	42.4
Yes	4184	51.2	4457	56.7	4640	57.6
Use an auditory device						
No	7934	97.1	7572	96.4	7758	96.2
Yes	233	2.9	282	3.6	302	3.8
Dental visits						
No	5558	68.1	5182	66.0	5598	69.5
≥1 visit	2609	31.9	2672	34.0	2462	30.5
Visited doctor						
No	1457	17.8	1788	22.8	2172	27.0
≥1 visit	6710	82.2	6066	77.2	5888	73.0
Would you say your health is…						
Excellent/very good	378	4.6	425	5.4	450	5.6
Good	1790	21.9	1753	22.3	1953	24.2
Fair	4010	49.1	3926	50.0	4031	50.0
Poor	1989	24.4	1750	22.3	1626	20.2
Multimorbidity						
Nonmultimorbidity	5513	67.5	5495	70.0	5649	70.0
Multimorbidity ≥2	2654	32.5	2359	30.0	2411	30.0
Health insurance coverage						
Insured	4706	57.6	4759	60.6	5179	64.3
Uninsured	3461	42.4	3095	39.4	2881	35.7

**Table 2:** Prevalence of self-reported diabetes by sociodemographic and health characteristics among Mexican older adults (MHAS 2015, 2018, and 2021).

**Variables**	**Self-reported diabetes**		
	**2015** **% (*n*) (95% CI)**	**2018** **% (95% CI)**	**2021** **% (95% CI)**
Age			
65–74 years	28.7 (1432) [27.5–30.0]	30.2 (1348) [28.8–31.5]	31.0 (1341) [29.6–32.3]
75–84 years	24.5 (602) [22.8–26.2]	27.0 (695) [25.3–28.7]	27.0 (762) [25.3–28.6]
≥85 years	15.2 (111) [12.7–17.9]	16.5 (134) [14.1–19.2]	17.5 (159) [15.1–20.1]
Gender			
Male	23.2 (880) [21.8–24.5]	24.1 (847) [22.7–25.5]	24.2 (844) [22.8–25.6]
Female	28.9 (1265) [27.6–24.5]	30.6 (1330) [29.2–32.0]	31.0 (1418) [29.6–32.3]
Marital status			
Without spouse/partner	26.2 (907) [24.7–27.6]	27.4 (902) [25.9–28.9]	27.4 (992) [25.9–28.8]
With spouse/partner	26.3 (1238) [25.0–27.5]	27.9 (1275) [26.7–29.2]	28.6 (1261) [27.3–30.0]
Place of residence			
Urban	27.5 (1821) [26.4–28.5]	29.1 (1835) [28.0–30.2]	29.4 (1894) [28.3–30.5]
Rural	21.0 (324) [19.0–23.1]	22.1 (342) [20.0–24.2]	22.7 (368) [20.7–24.8]
Years of education			
No education	24.1 (455) [22.2–26.1]	25.0 (419) [22.9–27.1]	25.1 (373) [22.9–27.3]
≤9 years	27.3 (1493) [26.2–28.5]	28.9 (1552) [27.7–30.0]	29.2 (1636) [28.0–30.4]
>9 years	24.0 (197) [21.2–27.0]	25.7 (206) [22.8–28.8]	26.1 (253) [23.4–28.9]
Currently smoker			
No	27.3 (2029) [26.2–28.3]	28.7 (2071) [27.7–29.7]	28.7 (2127) [27.7–29.7]
Yes	15.8 (733) [13.3–18.6]	16.5 (106) [13.8–19.6]	20.4 (135) [17.5–23.6]
Currently drinks alcohol			
No	26.9 (1544) [25.8–28.1]	23.1 (380) [21.1–25.2]	22.7 (397) [20.8–24.7]
Yes	20.2 (317) [18.3–22.2]	28.9 (1494) [27.6–30.1]	29.1 (1425) [27.8–30.4]
Never drank alcohol	32.4 (282) [29.3–35.6]	29.2 (303) [26.5–32.0]	31.1 (440) [28.7–33.5]
Wear glasses			
No	23.7 (945) [22.4–25.1]	25.5 (865) [24.0–26.9]	25.9 (887) [24.4–27.4]
Yes	28.7 (1200) [27.3–30.0]	29.4 (1312) [28.1–30.7]	29.6 (1375) [28.3–30.9]
Use an auditory device			
No	26.5 (2105) [25.6–27.5]	27.8 (2107) [26.8–28.8]	27.9 (2167) [26.9–28.9]
Yes	17.2 (40) [12.8–22.6]	24.8 (70) [20.1–30.2]	31.5 (95) [26.4–36.9]
Dental visits			
No	25.5 (1420) [24.4–26.7]	27.8 (1441) [26.6–29.0]	27.6 (1542) [26.4–28.7]
≥1 visit	27.8 (725) [26.8–29.5]	27.5 (736) [25.9–29.3]	29.2 (720) [27.4–31.1]
Visited doctor			
No	11.8 (172) [10.2–13.6]	13.5 (241) [11.9–15.1]	16.4 (355) [14.8–17.9]
≥1 visit	29.4 (1973) [28.3–30.5]	31.9 (1936) [30.7–33.1]	32.4 (1907) [31.2–33.5]
Would you say your health is…			
Excellent/very good	9.5 (36) [6.9–12.9]	12.2 (52) [9.4–15.7]	14.2 (64) [11.3–17.8]
Good	19.0 (340) [17.2–20.9]	20.9 (367) [19.0–22.9]	19.7 (385) [18.0–21.5]
Fair	28.1 (1127) [26.7–29.5]	29.9 (1176) [28.5–31.4]	31.7 (1278) [30.3–33.1]
Poor	32.3 (642) [30.2–34.3]	33.3 (582) [31.0–35.5]	32.9 (535) [30.6–35.2]
Multimorbidity			
Nonmultimorbidity	25.1 (1383) [23.9–26.2]	26.1 (1436) [24.9–27.3]	27.0 (1523) [25.8–28.1]
Multimorbidity ≥2	28.7 (762) [27.0–30.4]	31.4 (741) [29.6–33.3]	30.6 (739) [28.8–32.5]
Health insurance coverage			
Uninsured	28.5 (1340) [27.2–29.8]	30.7 (1461) [29.4–32.0]	30.6 (1584) [29.3–31.8]
Insured	23.3 (805) [21.9–24.7]	23.1 (716) [21.7–24.6]	23.5 (678) [22.0–25.1]

**Table 3:** Adjusted odds ratios from the logistic regression model for the self-reported diabetes and predictors variables among Mexican older people. MHAS 2021 (*n* = 8060).

**Variables**	**Crude OR (95% CI)**	**p**	**Adjust OR (95% CI)**	**p**
Gender				
Male	Reference		Reference	
Female	1.40 (1.26–1.54)	<0.001	1.39 (1.25–1.55)	<0.001
Age				
65–74 years	Reference		Reference	
75–84 years	0.82 (0.74–0.91)	<0.001	0.79 (0.71–0.89)	<0.001
≥85 years	0.47 (0.39–0.56)	<0.001	0.41 (0.33–0.52)	<0.001
Years of education				
≥10 years	Reference		Reference	
1–9 years	1.17 (1.00–1.36)	0.045	0.15 (0.97–1.36)	0.099
No education	0.94 (0.79–1.14)	0.582	1.03 (0.83–1.28)	0.759
Place of residence				
Urban	Reference		Reference	
Rural	0.70 (0.62–0.80)	<0.001	0.80 (0.69–0.93)	0.004
Wear glasses				
No	Reference		Reference	
Yes	1.20 (1.08–1.32)	<0.001	1.23 (1.16–1.30)	<0.001
Health insurance coverage				
Insured	Reference		Reference	
Uninsured	1.43 (1.28–1.58)	<0.001	1.35 (1.20–1.53)	<0.001
Multimorbidity				
Nonmultimorbidity	Reference		Reference	
Multimorbidity ≥2	1.19 (1.07–1.32)	0.001	1.13 (1.01–1.27)	0.028
Currently drinks alcohol				
No	Reference		Reference	
Yes	1.13 (1.03–1.25)	0.011	1.12 (1.00–1.25)	0.033

*Note:* Log likelihood = −4142.3457; Hosmer–Lemeshow test *p* =0.2381.

Abbreviations: CI: confidence interval, MHAS: Mexican Health and Aging Study 2021, OR: odds ratio.

## Data Availability

The data that support the findings of this study are freely available from the Mexican Health and Aging Study (MHAS) at https://enasem.org/Home/Index.aspx, reference number NIH R01AG018016.
